# Piclidenoson, an A3 adenosine receptor agonist, demonstrates clinical benefit in canine osteoarthritis: a pilot study

**DOI:** 10.3389/fvets.2026.1847566

**Published:** 2026-06-08

**Authors:** Rémy Hanf, Avital Bareket-Samish, Pierre Moulin, Frederic Manias, Jerome Seguela, Benjamin Baulez, Vincent Sutre, Jean-Baptiste Koch, Julien Salindre, Matthieu Dubruque, Dominique Tierny, Sari Fishman, Pnina Fishman

**Affiliations:** 1 Vetbiolix, Loos, France; 2 BioInsight Ltd, Binyamina, Israel; 3 FlorVets, Lessines, Belgium; 4 ManiaVet, Waudrez, Belgium; 5 Clinique Vétérinaire de Parme, Biarritz, France; 6 VPLUS, St Aubin De Blaye, France; 7 Clnique vétérinaire Danton, Fouquiereq-Lès-Lens, France; 8 Clnique Vétérinaire du Cèdre, Epron, France; 9 VPLUS, Mirambeau, France; 10 OCRvet dba Clinaxel, Lille, France; 11 Can Fite BioPharma Ltd, Ramat Gan, Israel

**Keywords:** canine, clinical study, mobility, osteoarthritis, pain, piclidenoson

## Abstract

**Objectives:**

Treatments for canine osteoarthritis, a degenerative joint disease associated with impaired mobility and pain, which is common in older dogs, are limited. The objective of this clinical study was to investigate the efficacy and safety of piclidenoson (an agonist at the A3 adenosine receptor) for canine osteoarthritis.

**Methods:**

Client-owned dogs with spontaneously developed osteoarthritis and a score ≥11 at baseline on the Liverpool Osteoarthritis in Dogs (LOAD) were randomized to PO piclidenoson at 100 or 500 μg/kg bid and followed for 90 days. The primary efficacy endpoint was change in LOAD score from baseline to Day 90.

**Results:**

The safety population included 21 dogs (100 μg/kg bid, *n* = 10; 500 μg/kg bid, *n* = 11). The efficacy population included 19 dogs (2 had no post-baseline LOAD score). Baseline characteristics were similar in the 2 groups. The change in LOAD from baseline to Day 90 was significant in the 500 μg/kg bid group (LS-mean LOAD score of 25.4 [SE, 3.8] at baseline vs. 18.6 [SE, 3.8] at Day 90, *p* < 0.001), whereas in the 100 μg/kg bid dose, the change was significant at Day 30 and lost significance by Day 90 (LS-mean LOAD score of 26.5 [SE, 4.5] at baseline vs. 23.3 [SE, 3.8] at Day 90, *p* = 0.109). Change in pain, as reflected in the visual analog scale (VAS) was also significant in the 500 μg/kg bid group (LS-mean VAS of 5.1 [SE, 0.7] at baseline vs. 2.9 [SE, 0.7] at Day 90, *p* < 0.001). Lameness/pain, evaluated by the veterinarian, were also significantly reduced from baseline to Day 90 in the 500 μg/kg bid group (*p* < 0.001). No serious adverse events deemed possibly- or probably-related to the study drug were reported.

**Clinical significance:**

Piclidenoson was well tolerated, and the findings suggest potential efficacy, thus supporting its further evaluation as a treatment for canine osteoarthritis.

## 1 Introduction

Osteoarthritis (OA), a non-infectious, degenerative joint disease, is among the most common diseases in older dogs. The prevalence of OA in dogs >8 years was found to be 39.2, 57.4, 35.9, and 36.4% for the shoulder, elbow, hip, and stifle, respectively (1). The primary risk factors for canine OA include older age, obesity, gonadectomy, breed, and predisposing joint conditions (e.g., cranial cruciate ligament rupture, joint dysplasia, and articular fracture) (2, 3).

The pathogenesis of OA in dogs involves damage to the extracellular matrix, apoptosis and depletion of chondrocytes, loss of cartilage, formation of osteophytes, and eventually, thickening of the joint capsule and loss of joint function (4). Data on the molecular pathways implicated in canine OA are limited. In humans, the data are available and show the involvement of multiple signaling pathways (e.g., Wnt/β-catenin, NF-κB), pro-inflammatory cytokines (e.g., interleukin [IL]-1 and IL-6), and associated factors (e.g., matrix metalloproteinases) (5). Notably, a study in dogs showed that the differences in gene expression between normal and OA cartilage were overall similar to those observed in humans (6).

Beyond nonpharmaceutical approaches (e.g., weight management, physiotherapy), the pharmaceutical options to treat OA in dogs are limited to the reduction of OA-associated pain by non-steroidal anti-inflammatory drugs (NSAIDs), the prostaglandin E2 EP4 receptor antagonist, grapiprant, and the recently FDA-approved canine-specific monoclonal antibody targeting the nerve growth factor (NGF), bedinvetmab (7, 8).

Piclidenoson (CF101, Can-Fite BioPharma Ltd., Israel) is a highly selective, orally bioavailable agonist at the A3 adenosine receptor (A3AR), a receptor involved in inflammation and cancer (9). Piclidenoson has demonstrated an excellent safety profile in over 2,000 patients across several autoimmune inflammatory diseases, and is currently investigated in a phase 3 trial as a treatment for moderate-to-severe plaque psoriasis (10). The protective role of A3R activation in OA is primarily supported by the progressive loss of cartilage and OA development observed in A3R-knockdown mice (11). Aligned with this hypothesis, all studies in animal models demonstrated the ability of piclidenoson to ameliorate the manifestation of arthritic diseases (12–14). Piclidenoson was shown to be very effective in relieving chronic pain, in an A3AR-mediated mechanism that does not involve the opioid receptor (15). More specifically, in a rat model of adjuvant-induced arthritis, oral administration of piclidenoson protected from degradation of joint cartilage. In a model of OA induced by surgical transection of the anterior cruciate ligament (ACLT model) in rats, piclidenoson prevented histologic alteration of joint cartilage and showed anti-nociceptive effects (16). The mechanism of joint preservation by piclidenoson in rats is not well understood. Piclidenoson exerts its anti-inflammatory activity by deregulating the Wnt/β-catenin and NF-κB signaling pathways and inhibiting the production of proinflammatory cytokines (e.g., tumor necrosis factor-α, IL-1, and IL-12) (9, 17). In a rat OA model, piclidenoson was shown to induce apoptosis of inflammatory cells that have infiltrated into the joints and act as a cartilage protective agent (14). Adenosine A3 receptor activation also exerts a chondroprotective effect by inhibiting the NLRP3 inflammasome-mediated pyroptosis pathway in chondrocytes (18). Piclidenoson downregulates 2 catabolic pathways involving cartilage degradation (RUNX2 and CaMKII) in human osteoarthritic articular chondrocytes (11). It protects primary pig articular chondrocytes from hypo-osmotic shock and prevents degradation of cartilage matrix induced by TNFɑ or hypo-osmotic shock (11).

Here we report the results of a non-comparative, randomized, open-label study investigating the efficacy potential and safety of piclidenoson in client-owned dogs with spontaneously developed OA.

## 2 Materials and methods

### 2.1 Study design

This proof-of-concept study was a multi-center, non-comparative, randomized, open-label study evaluating the efficacy and safety of 2 doses of piclidenoson in client-owned dogs with spontaneously developed OA. The study was conducted in 12 investigational sites in Belgium and France.

**Figure 1:**
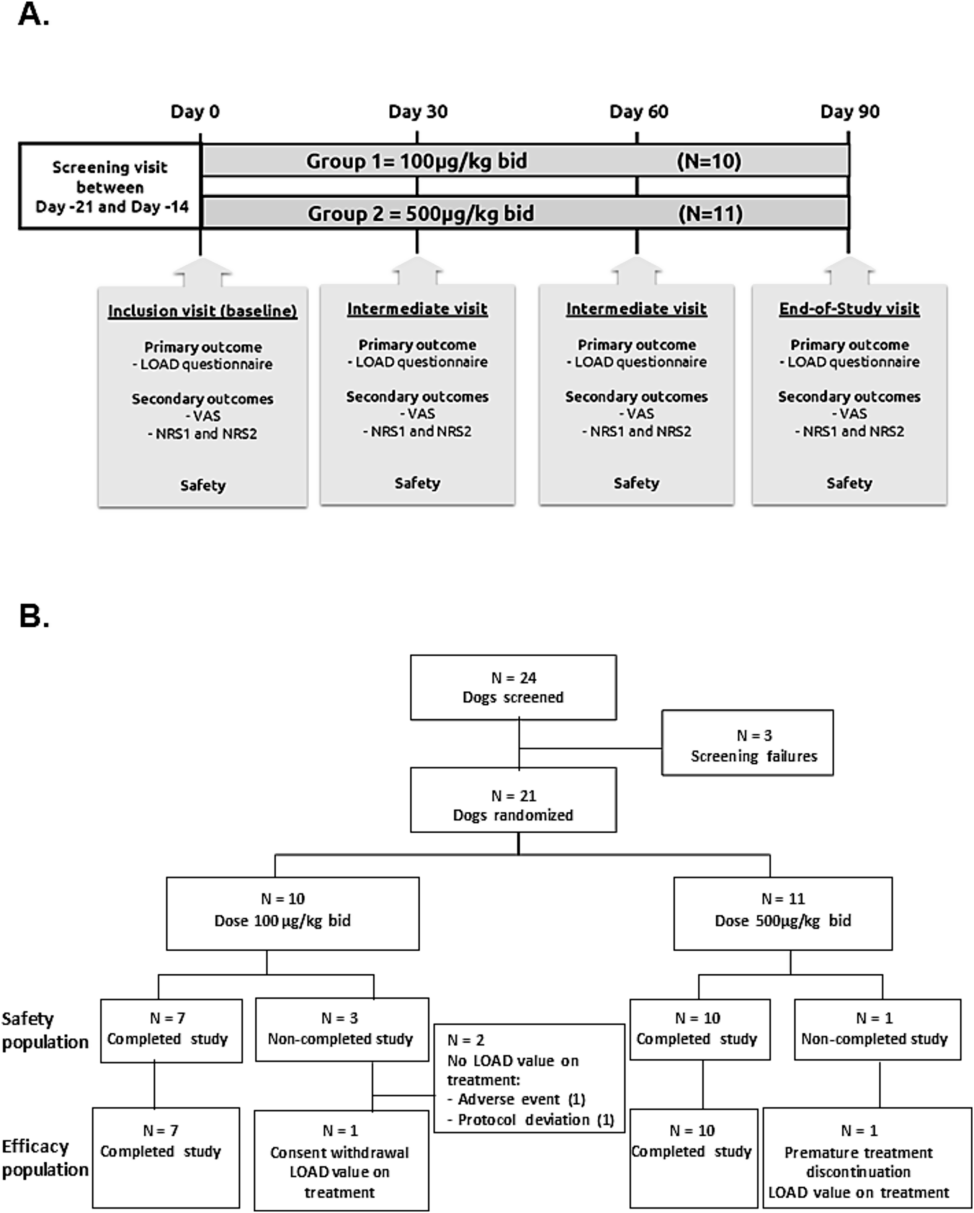
**(A)** Study design. **(B)** Patient disposition.

At inclusion visit (Day 0), the participating dogs were randomized (1:1) to PO piclidenoson at either 100 μg/kg bid or 500 μg/kg bid and were followed for 90 days. The assignment of the dogs to their treatment group was based on the order of entry into the study, regardless of the site. Both the owner and the veterinarian were aware of the treatment dose. The study included a total of 4 visits: Day 0 (inclusion visit), Day 30, Day 60, and Day 90 (Figure 1A). At each visit, the owner’s documentation of the Liverpool Osteoarthritis in Dogs [LOAD, a validated questionnaire with 13 questions that, when summed, result in an overall score of 0–52 with higher scores indicating increased OA severity (19, 20)] and pain evaluation using visual analog scale (VAS, 0–10 scale with increased values indicating increased pain) performed 1–2 days pre-visit were retrieved. In addition, at each visit, the veterinarian performed clinical evaluations of lameness using numerical rating score 1 (NRS1) and OA pain severity using numerical rating score 2 (NRS2; both NRS1 and NRS2 are unidimensional scales from 0 to 4 with increased scores indicating increased severity). For safety follow-up, blood samples were withdrawn, and any adverse events (AEs) occurring since the last visit were recorded. Throughout the study, dogs were kept in their usual housing, with their normal feeding and drinking routines.

The protocol was approved by the OCR Internal Ethics Committee (protocol # 22-VBX-01). All owners provided written informed consent for the participation of their dog.

### 2.2 Study doses

Study doses were selected by comparing pharmacokinetic data from healthy dogs and humans. Maximal plasma concentration (Cmax) and plasma exposure (AUCt) after repeated oral administration of 250 μg/kg piclidenoson in dogs were comparable to those obtained after repeated administration of a therapeutic oral dose of 2–3 mg in humans (Vetbiolix preclinical dossier, unpublished data).

### 2.3 Study population

The study included dogs that were >18 months, had a body condition score < 8 (9 point-scale), had a symptomatic unifocal or multifocal OA with ≥1 site of osteophyte on radiographic views, and LOAD score of ≥11 at baseline (Day 0). Key exclusion criteria included dogs that were pregnant, lactating, or intended for breeding; dogs with severe OA requiring concomitant treatment, presence of cranial cruciate ligament rupture, a history of orthopedic surgery, concurrent diseases (e.g., neurologic, hepatic, renal, cardiac, neoplasia, or musculoskeletal lesions), and prior treatment with methotrexate or NSAIDs in the preceding 2 weeks, bedinvetmab in the preceding 4 weeks, and mavacoxib or enflicoxib in the preceding 3 months. Dogs treated with short-acting, intermediate-acting, and long-acting corticosteroids in the preceding 2, 4, and 12 weeks, respectively, were excluded. Dogs under physical therapy for orthopedic indications in the preceding week were excluded, as were dogs treated with OA-modifying diet and/or food supplements, unless their administration had been regular during the month preceding the study and the diet/supplement were to be continued during the study. In addition, for breeds with a risk of *MDR1* gene mutation (Collie, Border Collie, Australian and English Shepherd, German Shepherd, Long-Haired Whippet, and Shetland Sheepdog), a genetic test was required, and dogs carrying the mutation were excluded.

Dogs were withdrawn before the conclusion of the study if the owner withdrew consent, was non-compliant, if the dog had severe concomitant disorder/disease, or the OA symptoms worsened such that a concomitant treatment including NSAIDs, corticosteroids, methotrexate, bedinvetmab, intra-articular administration of any treatment, physiotherapy, or any analgetic treatment except sedation for radiographic views included in the protocol was required.

### 2.4 Endpoints

The pre-specified primary efficacy endpoint was the effect of piclidenoson on reducing the severity of OA symptoms as reflected by the change in total LOAD score, as reported by the owner, between baseline and Day 90. Like other owner-reported questionnaires commonly used in interventional trials in dogs with OA, the LOAD score is a validated, owner-reported questionnaire designed to assess mobility and pain in dogs with OA. It has been extensively validated for reliability and responsiveness in both clinical and research settings (19). Other LOAD-associated endpoints included the effect of piclidenoson on the total LOAD score over time, and the proportion of dogs with a minimal clinically important difference (MCID) in total LOAD score [improvement by at least 4 points (21)] between baseline and Day 90.

Secondary efficacy endpoints included the effect of piclidenoson on reducing pain severity between baseline and Day 90 as reflected by the change in VAS as reported by the owner, and the proportion of dogs with MCID in VAS (defined as an improvement by at least 1 point).

Also, to confirm the owners’ evaluations on dog motility (LOAD) and OA pain (VAS), the effect of piclidenoson on reducing lameness and pain severity was independently scored by the veterinarian at each visit using NRS for lameness (NRS1) and pain (NRS2).

In addition, post-hoc exploratory efficacy analyses included intergroup comparisons for all evaluated efficacy endpoints.

The safety endpoints included the occurrence of AEs and serious AEs (SAEs) as well as evaluation of changes in hematological and biochemical parameters.

### 2.5 Statistical analysis

The safety or intention to treat (ITT) population included all dogs. The efficacy population included all treated dogs except those with no post-baseline LOAD value. In case of a missing value, the last post-baseline value was imputed to all subsequent visits (1 patient in the 100 μg/kg bid group). Descriptive statistics were used to summarize patient characteristics and safety.

For both the primary (LOAD) and secondary outcomes (VAS, NRS1, and NRS2) of the study, the principal statistical analyses were performed using mixed linear models. For analyses of within-group change of LOAD, VAS, NRS1, and NRS2 vs. baseline, estimates and *p* values were derived from mixed models with group and visit as fixed effects; visit X group interaction; and dog as a random effect. For the primary efficacy endpoint (change in LOAD between Day 90 and Day 0), a Wilcoxon matched-pairs signed-rank test was performed as a sensitivity analysis (non-normal distribution; Shapiro–Wilk test). For inter-group comparisons of change in LOAD and VAS in the 100 μg/kg bid and the 500 μg/kg bid groups, estimates and *p* values were derived using repeated measures mixed model with change from baseline as dependent variable; group and visit as fixed effects; visit X group interaction; visit as a repeated factor; baseline (Day 0) value as a covariate; and dog identified as the subject in the repeated statement with compound symmetry (CS) covariance structure. For comparison of the proportion of patients experiencing a clinically meaningful response at Day 90 in the 2 groups, statistical significance was assessed using Fisher’s exact test. Multiplicity was not taken into account in the analyses of secondary endpoints. Statistical analysis was performed using SAS (SAS Institute, NC, USA).

## 3 Results

### 3.1 Study dogs

Overall, 24 dogs were screened for this study between May 2023 and March 2024 in 7 veterinary clinics in Belgium and France. Twenty-one of them were randomized to receive PO piclidenoson at 100 μg/kg bid (*n* = 10) or 500 μg/kg bid (*n* = 11) and were included in the safety (ITT) population (Figure 1B). Seven dogs in the 100 μg/kg bid group and 10 dogs in the 500 μg/kg bid group completed the study. All dogs except 2 with no LOAD value on treatment (both in the 100 μg/kg bid group) were included in the efficacy population (Figure 1B).

The 2 treatment groups had overall comparable baseline characteristics with no major imbalance in age, sex ratio, gonadectomy rate, weight, and baseline LOAD, VAS, and NRS1/2 scores as shown in Table 1. The median (range) age in the 100 μg/kg bid and 500 μg/kg bid was 8.4 (3.3–14.3) and 10.0 (3.6–13.6) years, respectively. More male dogs were included in the 500 μg/kg bid than in the 100 μg/kg bid (55% vs. 30%, respectively). In both groups, the majority of dogs were spayed/neutered (80 and 54% in the 100 μg/kg bid and 500 μg/kg bid groups, respectively). The mean LOAD scores at baseline were comparable in the two groups (mean [SD] of 27.2 [10.2] and 25.4 [8.8], in the 100 μg/kg bid and 500 μg/kg bid groups, respectively).

**Table 1:** Population demographics at screening for the safety (ITT) population.

Characteristic	Piclidenoson 100 μg/kg bid *n* = 10	Piclidenoson 500 μg/kg bid *n* = 11
Age, years		
Mean (SD)	8.5 (3.2)	10.0 (3.0)
Median (range)	8.4 (3.3–14.3)	10.0 (3.6–13.6)
Sex, *n* (%)		
Female (total)	7 (70%)	5 (45%)
Female (intact)	1 (10%)	1 (9%)
Female (spayed)	6 (60%)	4 (36%)
Male (total)	3 (30%)	6 (55%)
Male (intact)	1 (10%)	4 (36%)
Male (neutered)	2 (20%)	2 (18%)
Weight, kg		
Mean (SD)	25.1 (16.5)	29.1 (7.3)
Median (range)	23.8 (4.0–55)	27.5 (20–42)
LOAD Score at baseline		
Mean (SD)	27.2 (10.2)	25.4 (8.8)
Median (range)	28 (12–45)	24 (11–42)
VAS Score at baseline		
Mean (SD)	4.7 (2.2)	5.1 (2.3)
Median (range)	5.0 (1.0–8.0)	5.0 (1.3–8.0)
NRS1 (lameness) at baseline		
Mean (SD)	1.8 (0.9)	1.7 (0.9)
Median (range)	1.5 (1–3)	2.0 (0–3)
NRS2 (pain) at baseline		
Mean (SD)	1.6 (0.7)	2.2 (1.0)
Median (range)	1.5 (1–3)	2.0 (0–4)

ITT, intention to treat; LOAD, Liverpool Osteoarthritis in Dogs; NRS, numerical rating score; SD, standard deviation; VAS, visual analog scale.

### 3.2 Efficacy (efficacy population)

**Figure 2:**
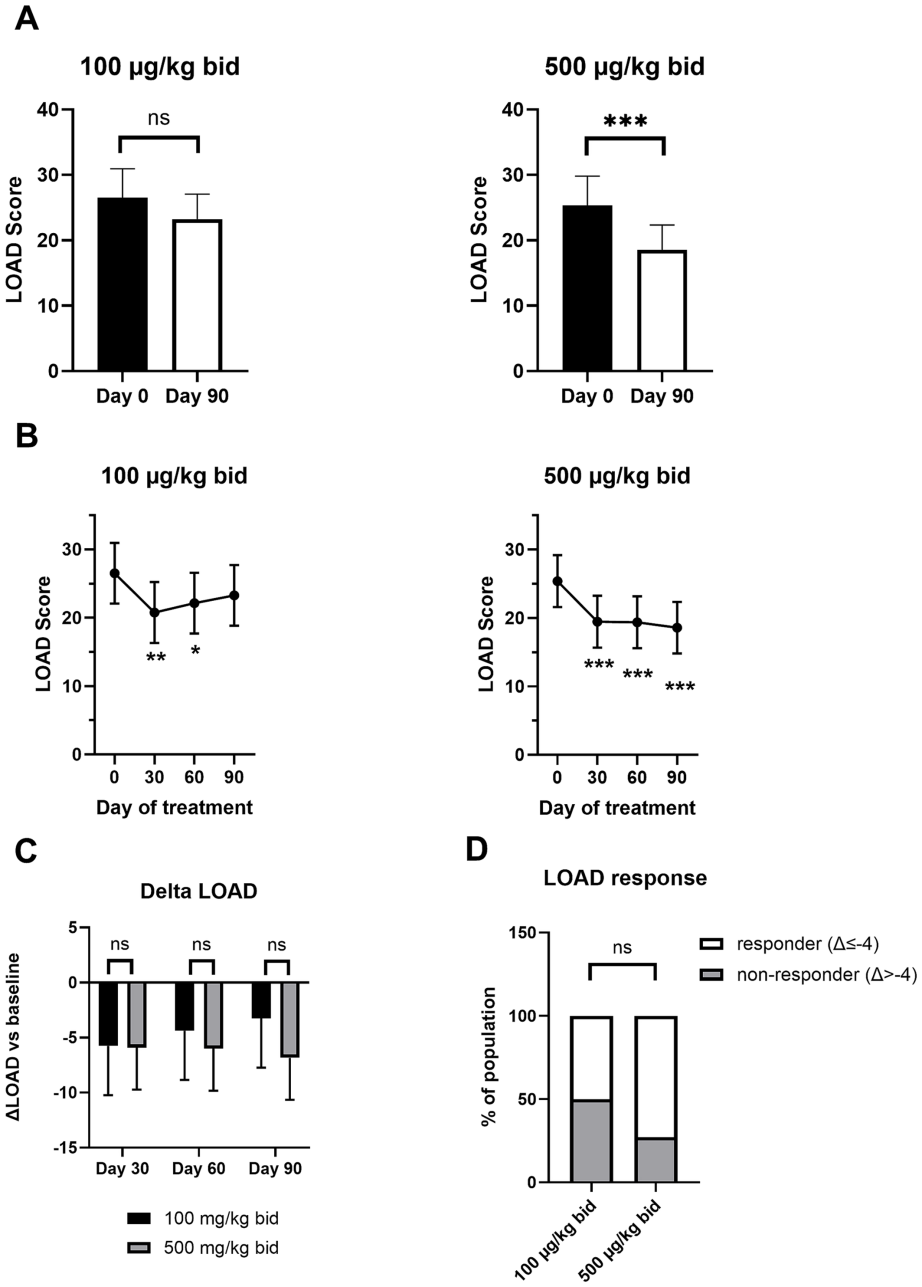
Changes from baseline in dog mobility as determined using the owner-reported LOAD score. **(A)** LOAD scores at baseline and Day 90 for the 100 μg/kg and 500 μg/kg groups. Data are presented as least-squares mean (LS-mean) ± SE with *p*-values derived from a linear mixed model. **(B)** Changes in LOAD score from Day 0 to Day 90 for both groups. Data are presented as LS-mean ± SE with *p*-values derived from a linear mixed model. **(C)** Effect size compared to baseline for both groups. Data are presented as LS-mean ± 95% CI with between-group comparison derived from a linear mixed model. **(D)** Proportion of dogs with or without a clinically meaningful change in LOAD score from Day 0 to Day 90. Statistical analysis was performed using Fisher’s exact test. ***p < 0.001, **p < 0.01 vs. baseline, ns denotes nonsignificant. Intergroup comparisons are indicated with bracket.

Evaluation of the primary efficacy endpoint demonstrated that the change in LOAD score from baseline to Day 90 was significant in the 500 μg/kg bid group with LS-mean (SE) LOAD score of 25.4 (3.8) at baseline vs. 18.6 (3.8) at Day 90 (*p* < 0.001). In the 100 μg/kg bid group, the change was nonsignificant with LS-mean (SE) LOAD score of 26.5 (4.5) at baseline vs. 23.3 (4.5) at Day 90 (*p* = 0.109) (Figure 2A).

Examining the effect of the treatment on LOAD score over time between Day 0 and Day 90 demonstrated a progressive decrease of LOAD score that reached significance at Day 30 in the 500 μg/kg bid groups; whereas, in the 100 μg/kg bid group, there was a significant effect at Day 30 that disappeared by Day 90 (Figure 2B). The trajectories of the change in LOAD score over time for the individual dogs in the 2 dose groups are presented in Supplementary Figure 1. Despite an apparent dose-dependent effect on LOAD, the change from baseline (delta LOAD) at each timepoint was not statistically significantly different between the 2 groups (Figure 2C). Also, at Day 90, 8 of 11 dogs (73%) in the 500 μg/kg bid group, and 4 of 8 dogs (50%) in the 100 μg/kg bid group experienced MCID in LOAD. The difference between the groups was not statistically significant (Figure 2D).

**Figure 3:**
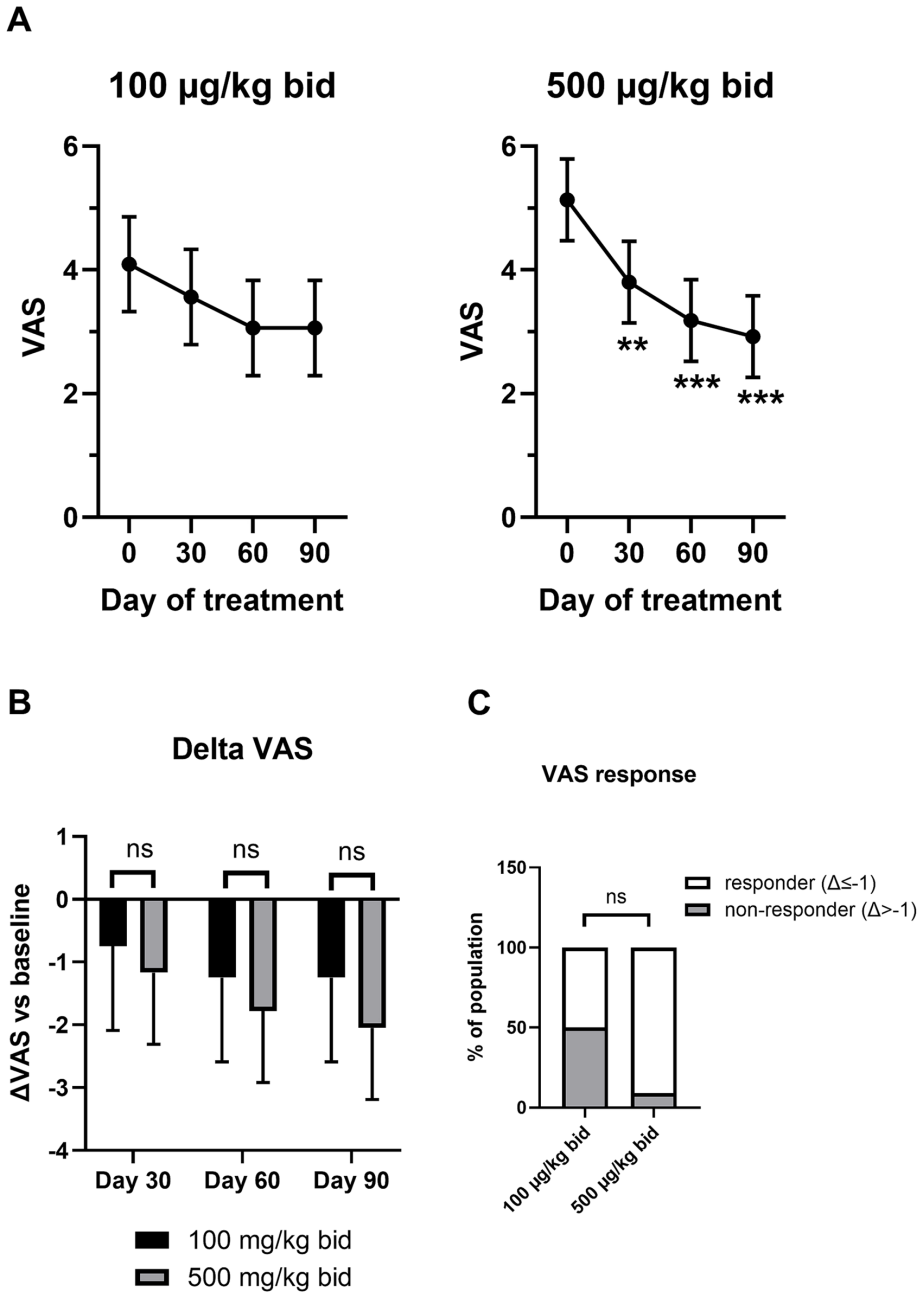
Changes from baseline in OA pain as determined using owner-reported VAS. **(A)** Changes in VAS from Day 0 to Day 90 for both groups. Data are presented as least-squares mean (LS-mean) ± SE with *p*-values derived from a linear mixed model. **(B)** Effect size compared to baseline for both groups. Data are presented as LS-mean ± 95% CI with between group comparison derived from a linear mixed model. **(C)** Proportion of dogs with or without a clinically meaningful change in VAS from Day 0 to Day 90. Statistical analysis was performed using Fisher’s exact test. ***p < 0.001, **p < 0.01 vs. baseline, ns denotes nonsignificant. Intergroup comparisons are indicated with bracket.

Assessment of the effects of the treatment on OA pain using VAS, as reported by the owner, showed an overall time-dependent improvement from baseline. In the 500 μg/kg bid group, a time-dependent decline in VAS was observed throughout the treatment period, with the difference vs. baseline reaching statistical significance from Day 30 forward (Figure 3A). In this group, LS-mean (SE) VAS was 2.9 (0.7) at Day 90 vs. 5.1 (0.7) at baseline (*p* < 0.001). In the 100 μg/kg bid group, a progressive decline in VAS was also observed without reaching significance throughout the treatment period (Figure 3A). In this group, LS-mean (SE) VAS was 3.1 (0.8) at Day 90 vs. 4.1 (0.8) at baseline (*p* = 0.07). Despite an apparent dose-dependent effect on VAS, comparison of the effect size between the 2 groups did not reach statistical significance at any of the time points (Figure 3B). In the 500 μg/kg bid group, 10 of 11 dogs (91%) and in the 100 μg/kg bid group, 4 of 8 dogs (50%) had a VAS improvement by more than 1 point between Day 0 and Day 90. This difference between the treatment groups was nonsignificant (Figure 3C).

**Figure 4:**
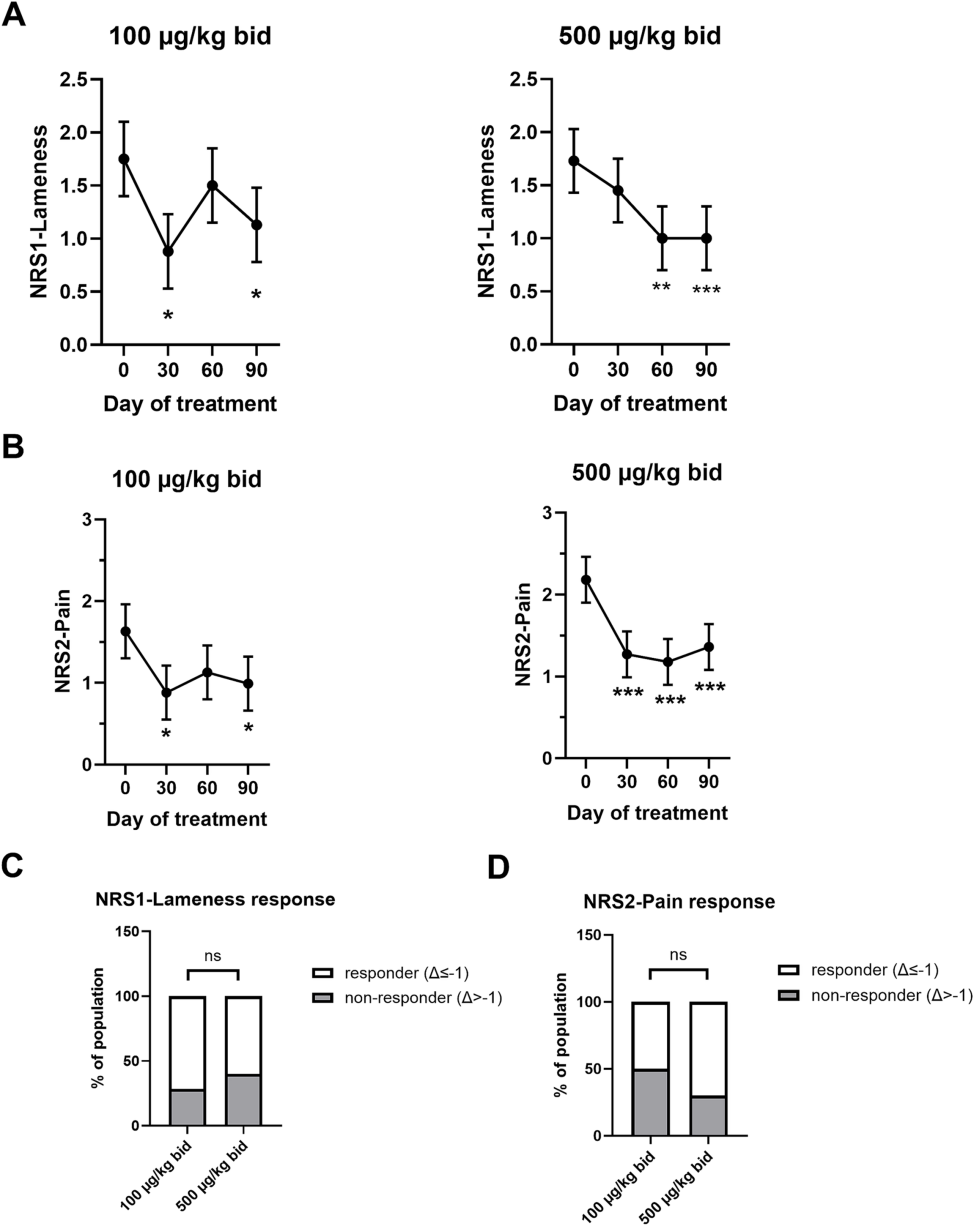
Changes from baseline in OA lameness (NRS1) and OA pain (NRS2) as determined by the veterinarian at clinical examination. **(A)** Changes in NRS1 from Day 0 to Day 90 for both groups. Data are presented as least-squares mean (LS-mean) ± SE with *p*-values derived from a linear mixed model as described in the methods. **(B)** Changes in NRS2 from Day 0 to Day 90 for both groups. Data are presented as LS-mean ± SE with *p*-values derived from a linear mixed model. **(C)** Proportion of dogs with or without reduction of NRS1 from Day 0 to Day 90. Statistical analysis was performed using Fisher’s exact test. **(D)** Proportion of dogs with or without reduction of NRS2 from Day 0 to Day 90. Statistical analysis was performed using Fisher’s exact test. ***p < 0.001, **p < 0.01 vs. baseline, ns denotes nonsignificant. Intergroup comparisons are indicated with bracket.

Evaluation of OA lameness (NRS1) and OA pain (NRS2) as determined by the veterinarian demonstrated improvement over time in the 500 μg/kg bid group, with the difference vs. baseline reaching statistical significance from Day 30 (NRS2) or Day 60 (NRS1) forward (Figures 4A,B). In the 100 μg/kg bid group, improvement over time in NRS1 and NRS2 was also observed, but was less consistent (Figures 4A,B). Comparison of the effect size between the 2 groups revealed no statistically significant difference between the groups at any of the timepoints, for both NRS1 and NRS2 (data not shown). In dogs with baseline NRS ≥ 1, in the 500 μg/kg bid group, response to treatment between Day 0 and Day 90 (ΔNRS ≤ −1) was reported in 6 of 10 (60%) dogs for NRS1 and in 7 of 10 (70%) dogs for NRS2. In the 100 μg/kg bid group, the respective values were (5/7, 71%) for NRS1 and (4/8, 50%) for NRS2. For both NRS1 and NRS2, the difference between the treatment groups was nonsignificant (Figures 4C,D).

### 3.3 Safety (ITT population)

The safety was evaluated in the 21 dogs that had received at least 1 dose of treatment. A total of 10 AEs in 7 dogs (3 dogs in the 100 μg/kg bid group and 4 dogs in the 500 μg/kg bid group) were reported during the study (Table 2). The causal relationships to the treatment were classified by the investigators as “not related” or “not assessable” for 7 AEs, whereas 3 AEs were judged as “possibly” or “probably” related to the treatment. Two not-treatment-related SAEs were reported: one case of newly diagnosed fibrosarcoma in the 500 μg/kg bid group and one case of renal insufficiency (suspicion of poisoning in the neighborhood) that led to euthanasia of the dog in the 100 μg/kg bid group. All other AEs observed were mild or moderate and resolved quickly with veterinary care. Additionally, an AE involving a skin reaction that necessitated corticosteroid treatment occurred, and led to the withdrawal of a dog from the study after 10 days of treatment. This AE resolved quickly following discontinuation of the experimental treatment and medical intervention. Hematological and biochemical parameters remained within the normal limits for all dogs throughout the study period.

**Table 2:** Summary of adverse events by severity.

			Piclidenoson 100 μg/kg bid *n* = 10		Piclidenoson, 500 μg/kg bid *n* = 11		
System organ class Preferred term	AE/SAE	Severity	Number of animals *n* (%)	Number of events	Number of animals *n* (%)	Number of events	Relation to treatment
Any adverse event			3 (30.0)	3	4 (36.4)	7	
Behavioral disorders			0 (0)	0	1 (9.1)	1	
Biting-aggression	AE	Moderate	0 (0)	0	1 (9.1)	1	Not related
Digestive tract disorders			0 (0)	0	1 (9.1)	2	
Diarrhea, Dysorexia	AE	Moderate	0 (0)	0	1 (9.1)	1	Probable
Diarrhea, Enteritis	AE	Moderate	0 (0)	0	1 (9.1)	1	Possible
Investigations			1 (10.0)	1	0 (0)	0	
Haematuria	AE	Mild	1 (10.0)	1	0 (0)	0	Not assessable
Neoplasia			0 (0)	0	1 (9.1)	1	
Fibrosarcoma	SAE	Severe	0 (0)	0	1 (9.1)	1	Not related
Neurological disorders			0 (0)	0	1 (9.1)	1	
Horner’s syndrome	AE	Moderate	0 (0)	0	1 (9.1)	1	Not assessable
Renal and urinary disorders			1 (10.0)	1	1 (9.1)	1	
Cystitis	AE	Moderate	0 (0)	0	1 (9.1)	1	Not assessable
Renal insufficiency	SAE	Severe	1 (10.0)	1	0 (0)	0	Not related
Skin and appendages disorders			1 (10.0)	1	0 (0)	0	
Dermatitis and eczema	AE	Moderate	1 (10.0)	1	0 (0)	0	Possible
Systemic disorders			0 (0)	0	1 (9.1)	1	
Anorexia	AE	Mild	0 (0)	0	1 (9.1)	1	Not assessable

Each AE is counted once per animal within system organ class and preferred term, at the highest reported severity.

AE, adverse event; SAE, serious adverse event.

## 4 Discussion

The findings of this exploratory non-comparative, randomized study suggest that a dose of 500 μg/kg bid piclidenoson was safe and showed preliminary efficacy signals in improving OA symptoms, as evaluated by the owner (dog mobility and pain) or by the veterinarian at a clinical examination (lameness and pain). The results demonstrated improvement with 500 μg/kg bid piclidenoson that increased over time, such that the effects became statistically significant in all evaluated measures from Day 30 to 60 of treatment. In contrast, at 100 μg/kg bid, not all evaluated measures demonstrated significant improvements over time, although numerical improvements were observed. Also, for the LOAD endpoint, the findings that at 100 μg/kg bid, piclidenoson showed significant improvements by Day 30 that disappeared by Day 90, suggest that longer treatment may negate the positive effects of 100 μg/kg bid. Thus, although the results of this study are encouraging, the dose dependency and the optimal duration of treatment remain to be further explored.

The results of the current study cannot be directly compared to those from the pivotal trials evaluating the grapiprant tablets or the bedinvetmab injections in canine OA, as the assessment tools were different from those used here (7, 22). Notably, the current findings are consistent with a randomized placebo-controlled study of an investigational caninised anti-NGF antibody (NV-01), which did use LOAD in addition to other assessment tools. This NV-01 study demonstrated significant improvements in LOAD scores over time (from Day 0 to Day 14 and from Day 0 to Day 28), in the investigational group but not in the control group (23). In addition to the difference in assessment tools, our study design also differs from that of the pivotal trials for grapiprant and bedinvetmab, as these trials had a placebo (grapiprant) or an active-control arm (bedinvetmab where the control group received the NSAID meloxicam) (7, 22). Both the grapiprant and the bedinvetmab trials demonstrated effectiveness of the evaluated drug as defined in their respective protocols (7, 22). In the bedinvetmab study, bedinvetmab and meloxicam were equally effective in managing OA-related pain; however, bedinvetmab was associated with fewer AEs. Our findings suggest that piclidenoson could be a safe and effective treatment option for canine OA. The favorable safety profile observed with piclidenoson in this study is consistent with the clinical studies in humans evaluating piclidenoson for autoimmune inflammatory diseases (e.g., rheumatoid arthritis and psoriasis), where a total of over 2,000 patients received piclidenoson (24–27). Piclidenoson, similar to grapiprant and unlike bedinvetmab, is administered orally, which may be more convenient for the owners/caretakers of the dogs. Unlike bedinvetmab, which only targets the pain aspect of OA, piclidenoson and grapiprant also target the inflammatory component, and thus the underlying OA pathogenesis. Notably, grapiprant, similar to other NSAIDs and unlike piclidenoson, is characterized by gastrointestinal AEs (7).

The safety profile of piclidenoson in dogs, as suggested by the current findings, and its administration mode support its long-term use, thus facilitating its use in the early stages of OA to prevent irreversible joint damage. The current study included all dogs regardless of baseline LOAD score; however, focusing on dogs with a baseline LOAD score of 11–35 may be useful, as this range is sufficiently high to allow the effects of the drug to be observed, yet sufficiently low to ensure that any damage to the joints caused by the OA before the study is at least partially reversible.

This was an exploratory proof-of-concept study designed to evaluate the active dosage of piclidenoson for canine OA, to assess the safety profile of this drug in dogs, and to explore its potential efficacy as a treatment for canine OA. This study has several limitations, including an open-label non-comparative design without a placebo or an active-control comparator. The lack of a control group limited our ability to draw conclusions regarding the efficacy of the drug and to estimate the effect size. Also, the small sample size limited the statistical power and did not allow a formal demonstration of dose-dependency. Thus, our ability to draw definitive conclusions was limited. Lastly, both the LOAD questionnaire and VAS are subjective owner-reported outcomes, which are based on the owners’ perception of the dogs’ disability. This subjectivity may be a limitation, particularly in a non-comparative, open-label design, with the owners being aware of the dose received (19, 20). To mitigate the risk of overinterpretation, clinical examination performed by the veterinarian at each visit confirmed the owner’s evaluation.

In conclusion, this exploratory study showed that the adenosine A3 receptor agonist, piclidenoson, was well tolerated and demonstrated preliminary efficacy signals. Thus, the findings support the continued evaluation of the optimal dosing/treatment duration of piclidenoson and its efficacy as a treatment for canine OA. Larger randomized, double-blind, placebo-controlled or active-controlled trials are warranted.

## Data Availability

The raw data supporting the conclusions of this article will be made available by the authors without undue reservation.
