# Supplementary material for: Piclidenoson, an A3 adenosine receptor agonist, demonstrates clinical benefit in canine osteoarthritis: a pilot study
Source: Front Vet Sci. 2026 Jun 8;13:1847566. doi: 10.3389/fvets.2026.1847566 (PMC13285384; doi:10.3389/fvets.2026.1847566)
Supplement: Supplementary file 1 [file Data_Sheet_1.pdf]

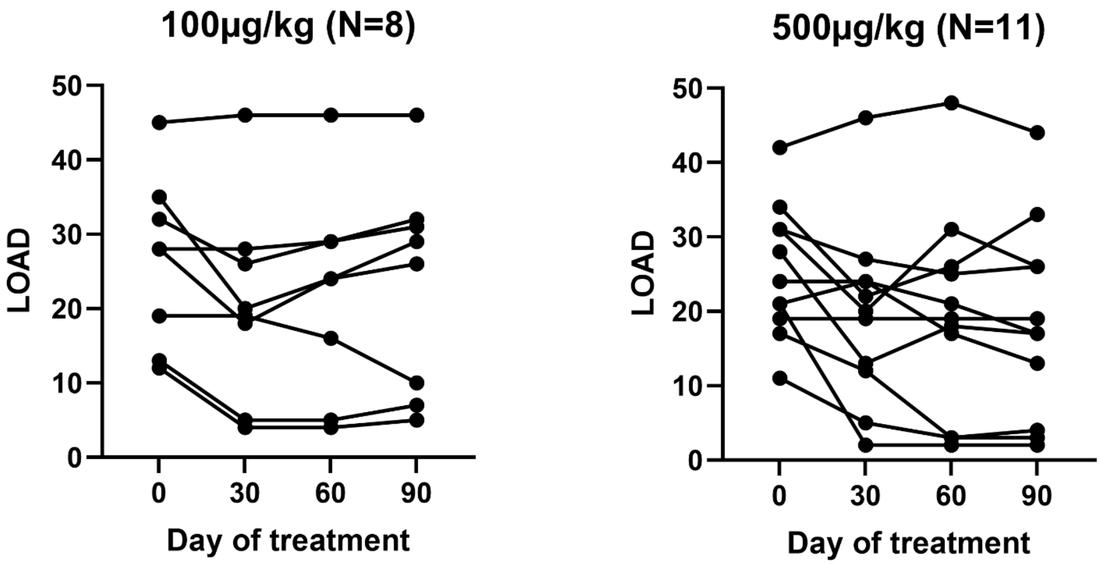

Supplementary Material

Supplementary Figure 1. Owner-reported LOAD scores from Day 0 through Day 90 for the individual dogs in the piclidenoson 100 µg/kg bid group and in the 500 µg/kg bid group.
